# Early discontinuation of PD-1 blockade upon achieving a complete or partial response in patients with advanced melanoma: the multicentre prospective Safe Stop trial

**DOI:** 10.1186/s12885-021-08018-w

**Published:** 2021-03-25

**Authors:** E. E. A. P. Mulder, K. de Joode, S. Litière, A. J. ten Tije, K. P. M. Suijkerbuijk, M. J. Boers-Sonderen, G. A. P. Hospers, J. W. B. de Groot, A. J. M. van den Eertwegh, M. J. B. Aarts, D. Piersma, R. S. van Rijn, E. Kapiteijn, G. Vreugdenhil, F. W. P. J. van den Berkmortel, E. Oomen-de Hoop, M. G. Franken, B. Ryll, P. Rutkowski, S. Sleijfer, J. B. A. G. Haanen, A. A. M. van der Veldt

**Affiliations:** 1 Department of Medical Oncology, Erasmus Medical Centre Cancer Institute, Rotterdam, The Netherlands; 2 Department of Surgical Oncology, Erasmus Medical Centre Cancer Institute, Rotterdam, The Netherlands; 3 European Organisation for Research and Treatment of Cancer (EORTC), Brussels, Belgium; 4 Department of Internal Medicine, Amphia Hospital, Breda, The Netherlands; 5 Department of Medical Oncology, University Medical Centre Utrecht Cancer Centre, Utrecht, The Netherlands; 6 Department of Medical Oncology, Radboud University Medical Centre, Nijmegen, The Netherlands; 7 Department of Medical Oncology, University Medical Centre Groningen, Groningen, The Netherlands; 8 Department of Medical Oncology, Isala Oncological Centre, Zwolle, The Netherlands; 9 Department of Medical Oncology, Amsterdam University Medical Centre – location VU, Amsterdam, The Netherlands; 10 Department of Medical Oncology, Maastricht University Medical Centre +, Maastricht, The Netherlands; 11 Department of Internal Medicine, Medisch Spectrum Twente, Enschede, The Netherlands; 12 Department of Internal Medicine, Medical Centre Leeuwarden, Leeuwarden, The Netherlands; 13 Department of Medical Oncology, Leiden University Medical Centre, Leiden, The Netherlands; 14 Department of Internal Medicine, Máxima Medical Centre, Veldhoven, The Netherlands; 15 Department of Internal Medicine, Zuyderland Medical Centre, Sittard-Geleen, The Netherlands; 16 Institute for Medical Technology Assessment, Erasmus School of Health Policy & Management, Erasmus University Rotterdam, Rotterdam, The Netherlands; 17 Melanoma Patient Network Europe, Uppsala, Sweden; 18 Department of Soft Tissue/Bone Sarcoma and Melanoma, Maria Sklodowska-Curie National Research Institute of Oncology, Warsaw, Poland; 19 Department of Medical Oncology, Netherlands Cancer Institute-Antoni van Leeuwenhoek Hospital, Amsterdam, The Netherlands; 20 Department of Radiology & Nuclear Medicine, Erasmus Medical Centre Cancer Institute, Rotterdam, The Netherlands

**Keywords:** Melanoma, Advanced and metastatic, PD-1 blockade, Response (complete or partial), Health-related quality of life

## Abstract

**Background:**

The introduction of programmed cell death protein 1 (PD-1) blockers (i.e. nivolumab and pembrolizumab) has significantly improved the prognosis of patients with advanced melanoma. However, the long treatment duration (i.e. two years or longer) has a high impact on patients and healthcare systems in terms of (severe) toxicity, health-related quality of life (HRQoL), resource use, and healthcare costs. While durable tumour responses have been observed and PD-1 blockade is discontinued on an individual basis, no consensus has been reached on the optimal treatment duration. The objective of the Safe Stop trial is to evaluate whether early discontinuation of first-line PD-1 blockade is safe in patients with advanced and metastatic melanoma who achieve a radiological response.

**Methods:**

The Safe Stop trial is a nationwide, multicentre, prospective, single-arm, interventional study in the Netherlands. A total of 200 patients with advanced and metastatic cutaneous melanoma and a confirmed complete response (CR) or partial response (PR) according to response evaluation criteria in solid tumours (RECIST) v1.1 will be included to early discontinue first-line monotherapy with nivolumab or pembrolizumab. The primary objective is the rate of ongoing responses at 24 months after discontinuation of PD-1 blockade. Secondary objectives include best overall and duration of response, need and outcome of rechallenge with PD-1 blockade, and changes in (serious) adverse events and HRQoL. The impact of treatment discontinuation on healthcare resource use, productivity losses, and hours of informal care will also be assessed. Results will be compared to those from patients with CR or PR who completed 24 months of treatment with PD-1 blockade and had an ongoing response at treatment discontinuation. It is hypothesised that it is safe to early stop first-line nivolumab or pembrolizumab at confirmed tumour response while improving HRQoL and reducing costs.

**Discussion:**

From a patient, healthcare, and economic perspective, shorter treatment duration is preferred and overtreatment should be prevented. If early discontinuation of first-line PD-1 blockade appears to be safe, early discontinuation of PD-1 blockade may be implemented as the standard of care in a selected group of patients.

**Trial registration:**

The Safe Stop trial has been registered in the Netherlands Trial Register (NTR), Trial NL7293 (old NTR ID: 7502), https://www.trialregister.nl/trial/7293. Date of registration September 30, 2018.

## Background

Cutaneous melanoma originates from melanocytes [1]. Although melanoma has a lower incidence than other types of skin cancer, melanoma is responsible for the majority of skin cancer-related deaths worldwide, and its incidence is increasing [2, 3]. The life expectancy of melanoma patients is mainly determined by disease stage, and patients with advanced-stage disease have a significantly worse prognosis [4]. Since the introduction of immune checkpoint inhibitors (ICIs) and targeted therapies, the prognosis of patients with advanced (i.e. irresectable stage IIIC) and metastatic (i.e. stage IV) melanoma has improved significantly [5–7]. Nowadays, first-line monotherapy with the programmed cell death protein 1 (PD-1) blockers nivolumab (Opdivo®) or pembrolizumab (Keytruda®) is administered most frequently in patients with advanced and metastatic melanoma [8–10].

In the phase III registration trials [5, 11], PD-1 blockers were usually administered for up to two years or even longer [12, 13]. Interestingly, durable tumour responses have been observed after early discontinuation (< 2 years) of PD-1 blockade [14–16], not only in patients who achieved a complete response (CR) [16] but also in patients with a partial response (PR) or stable disease (SD) [17, 18]. In patients who discontinued PD-1 blockade early because of adverse events (AEs), outcomes were not compromised and tumour responses were ongoing at > 5 years after treatment commencement [5, 19–22]. Therefore, early discontinuation of PD-1 blockade is considered feasible [23], in particular since rechallenge with PD-1 blockade induces overall response rates of up to 90% in patients with progressive disease (PD) after prior discontinuation of PD-1 blockade [24]. As pre-specified criteria for (early) discontinuation of PD-1 blockade were lacking in retrospective analyses [25, 26], it is not yet known which patients can safely discontinue PD-1 blockade at an earlier time point. Based on the median time to objective response of approximately 3 months in patients with advanced melanoma [11, 27], the preferred treatment duration of PD-1 blockade is considered to be at least 3–6 months [25].

Shorter treatment duration with PD-1 blockade would yield several major advantages since the long treatment duration of PD-1 blockade has a high impact on patients, healthcare systems, and healthcare costs [28].. First, treatment with PD-1 blockade is associated with a broad spectrum of AEs (e.g. pneumonitis, colitis, nephritis, and endocrinopathies), which can develop rapidly, severely, and can be, although rare, even fatal [29–32]. Some of these AEs can persist lifelong (e.g. hypothyroidism, type 1 diabetes). Although the incidence of these immune-related AEs is not associated with the dose of PD-1 blockade, the incidence gradually increases with treatment duration [33]. Second, PD-1 blockers are intravenously (IV) administered at the outpatient clinic regularly with intervals of 2, 3, 4, or 6 weeks, which may significantly affect patients’ health-related quality of life (HRQoL), productivity, and time of informal caregivers. Third, a treatment duration of ≥ 2 years is associated with high costs for expensive drugs, IV treatment administration, personnel, and management of treatment-related AEs.

From a patient, healthcare, and economic perspective, shorter treatment duration is obviously preferred and overtreatment should be prevented. In current clinical practice, an increasing number of physicians discontinue treatment on an individual basis in patients achieving tumour response, in case of severe toxicity, or on patients’ request [25]. However, substantial evidence about the safety of early discontinuation of PD-1 blockade is lacking. Therefore, a nationwide prospective interventional study has been initiated to generate evidence on early discontinuation of first-line PD-1 blockade in patients with advanced and metastatic melanoma. For feasible implementation in clinical practice, the study has been designed according to patients’ wishes and procedures of daily clinical practice.

## Methods

### Objectives

The primary objective is to evaluate the rate of ongoing response in patients with advanced and metastatic melanoma who early discontinue first-line monotherapy with nivolumab or pembrolizumab upon achieving CR or PR (i.e. before two years of treatment). Secondary objectives include disease outcome, AEs, and HRQoL. In addition, the impact of early PD-1 blockade discontinuation on productivity (paid and unpaid work), healthcare resources, and hours of informal care will be measured.

### Study design

#### Design

The Safe Stop trial is a nationwide, multicentre, prospective, single-arm, interventional trial in the Netherlands. According to the KEYNOTE-006 trial, the (maximum) treatment duration of PD-1 blockade has been set at two years in the Netherlands [5]. The current protocol has been developed at the 19th European Cancer Organisation - American Association for Cancer Research - European Organisation for Research and Treatment of Cancer – European Society for Medical Oncology (ECCO-AACR-EORTC-ESMO) Workshop on Methods in Clinical Cancer Research with the support of a biostatistician, melanoma surgeon, medical oncologist, and patient advocate from ESMO. As patients usually have access to long treatment duration (2 years or longer) with PD-1 blockade, randomisation to early discontinuation was considered not feasible in this setting as the sample size for a randomised non-inferiority study would have been much higher and patients may refuse the randomisation.

#### Participation sites

In the Netherlands, all patients with advanced and metastatic melanoma are treated in one of the 14 designated Dutch melanoma centres. The current nationwide study is executed in all 14 Dutch melanoma centres, which have close collaborations within the WIN-O (Dutch Working group on Immunotherapy of Oncology) and DMTR (Dutch Melanoma Treatment Registry): Amphia Hospital, Breda; Amsterdam University Medical Centres – location VU, Amsterdam; Antoni van Leeuwenhoek - Netherlands Cancer Institute, Amsterdam; Erasmus Medical Centre Cancer Institute, Rotterdam; Isala Clinics, Zwolle; Leiden University Medical Centre, Leiden; Maastricht University Medical Centre+, Maastricht; Máxima Medical Centre, Veldhoven; Medical Centre Leeuwarden, Leeuwarden; Medical Spectrum Twente, Enschede; Radboud University Medical Centre, Nijmegen; University Medical Centre Groningen, Groningen; University Medical Centre Utrecht, Utrecht; Zuyderland Medical Centre, Sittard-Geleen. In December 2018, the first site (Erasmus Medical Centre Cancer Institute) opened for inclusion. At the moment, all sites are open for inclusion.

#### Overview of current protocol

**Fig. 1:**
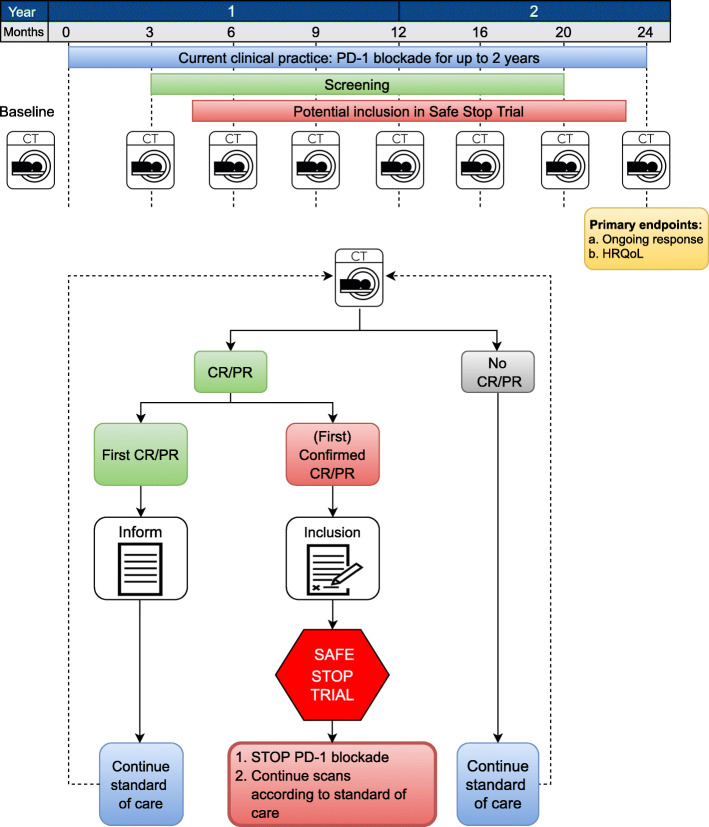
Timeline & flowchart of study assessments Safe Stop trial for first two years from start of PD-1 blockade. Abbreviations: CR, complete response; CT, computed tomography; HRQoL, Health-related quality of life; PD-1 blockade, programmed cell death protein 1 blockade; PR; partial response

For response evaluation after the initiation of PD-1 blockade, diagnostic computed tomography (CT) and/or magnetic resonance imaging (MRI) are required every 12 ± 1 weeks. Patients can participate after confirmed response (CR or PR) according to response evaluation criteria in solid tumours (RECIST) v1.1 [34]. When patients achieve their first CR or PR upon first-line PD-1 blockade, written patient information can be provided (see Fig. 1 for timeline and study assessments). At 6–12 (+ 1) weeks after the first documentation of CR and at 12 (±1) weeks after the first documentation of PR, tumour response needs to be confirmed according to RECIST v1.1. Patients with a confirmed response and willing to discontinue first-line PD-1 blockade are eligible for inclusion and subsequently discontinue treatment up to 6 (+ 1) weeks after first confirmation of response (CR or PR). In case patients experience PD after trial enrolment, PD-1 blockade will be restarted. Another salvage therapy is allowed at the discretion of the treating physician.

#### Study population

Patients are eligible for this study when the following inclusion criteria are met:

- age ≥ 18 year
- advanced or metastatic cutaneous melanoma
- current monotherapy with first-line nivolumab or pembrolizumab for advanced or metastatic melanoma; previous systemic treatment, including immunotherapy, in (neo) adjuvant setting for resectable melanoma is allowed
- documented diagnostic CT or MRI at the start of PD-1 blockade with nivolumab or pembrolizumab
  - for patients with CR on a diagnostic CT at response evaluation, a low-dose CT (i.e. usually included for positron emission tomography [PET] using fluorine-18 fluorodeoxyglucose [^18^F-FDG]) is allowed at baseline
  - for patients with PR on a diagnostic CT at response evaluation, a low-dose CT (i.e. usually included for ^18^F-FDG PET) is allowed at baseline if sufficient target lesions are measurable for response evaluation according to RECIST v1.1 criteria [34]; in this specific case, the sponsor should be consulted
- documented tumour response evaluation every 12 ± 1 weeks according to RECIST v1.1 [34] using a diagnostic CT and/or MRI as per standard clinical practice
- having confirmed CR (with an interval of 6–12 [+ 1] weeks after first documentation) or an ongoing PR (with an interval of 12 [±1] weeks after first documentation) according to RECIST v1.1 [34] using a diagnostic CT and/or MRI
- presence of MRI brain for the screening of brain metastases (prior to first start or discontinuation of PD-1 blockade)
- planned and willing to discontinue nivolumab or pembrolizumab within 6 (+ 1) weeks after first confirmation of CR or PR and within 2 years from initiation of treatment
- signed and dated informed consent form

A potential subject who meets the following criterion will be excluded from participation in this study:

- concomitant systemic therapies with other anti-cancer agents, e.g. BRAF-inhibitor, anti-CTLA4 (e.g. ipilimumab), or other PD-1 blockade than nivolumab or pembrolizumab

#### Follow-up

After confirmed CR/PR and subsequent early discontinuation of PD-1 blockade, clinical evaluation (visits, laboratory measurements, and diagnostic CT) will be performed every 12 (±1) weeks, according to the standard of care in the Netherlands (see Table 1). At inclusion and thereafter, patients will be asked to complete HRQoL and resource use questionnaires. Patient-reported outcomes of non-preference-based disease-specific and preference-based generic HRQoL, fear, productivity, informal care, and healthcare resource use outside the hospital will be assessed using Functional Assessment of Cancer Therapy Melanoma (FACT-M) [35], EuroQoL Health Utilities Index (EQ-5D, version 5 L) [36, 37], cancer worry scale (CWS) [38], and Resources Utilization Questionnaire Melanoma (RUQ-M) by the Institute for Medical Technology Assessment (iMTA) [39]. After the first year, the interval between the visits will increase. In case patients experience PD according to RECIST v1.1, PD-1 blockade will be restarted. Another salvage therapy is allowed at the discretion of the treating physician. For all included patients, data will be collected until the end of the follow-up period of the study, death, withdrawal, or other reasons for early discontinuation (whichever comes first).

**Table 1:**
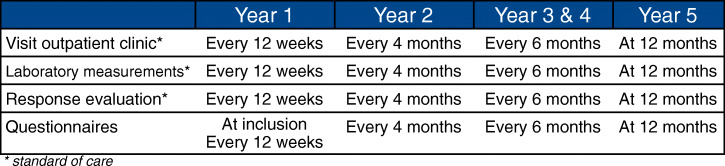
Follow-up scheme after confirmed CR/PR and subsequent discontinuation of PD-1 blockade

Abbreviation: PD-1 blockade, programmed cell death protein 1 blockade

## Study endpoints and analyses

### Primary endpoint

The primary endpoint of this study is the rate of ongoing response according to RECIST v1.1 [34] at 24 months (from first start of treatment with PD-1 blockade).

#### Analysis of the primary endpoint

The rate of ongoing response at 24 months (or equivalently progression-free survival [PFS] from start of treatment) will be estimated using the Kaplan-Meier method and its one-sided 95% confidence interval (CI) (as the lower boundary of a two-sided 90% CI). These results will be compared with the response rate of a well-defined historical cohort of first-line treatment with pembrolizumab in advanced melanoma (KEYNOTE-006) [5]. Among all patients who had a CR or PR in the KEYNOTE-006, 39% of patients completed 24 months of treatment and had an ongoing response at treatment discontinuation. A 29% ongoing response rate at 24 months after treatment start in patients who early discontinue treatment is considered acceptable, as the current protocol will include less selected patients from clinical practice and patients will have the option to restart treatment with nivolumab or pembrolizumab at PD, thereby potentially achieving a second tumour response. The study will be declared positive for the primary endpoint if the one-sided 95% CI is higher than 29%. The two-sided 90% CI will be constructed using the generalised Brookmeyer and Crowley method based on a g-transformed CI [40, 41].

### Secondary endpoints

The secondary endpoints include best overall response, duration of response after discontinuation of PD-1 blockade, the need and outcome of rechallenge with PD-1 blockade, and changes in SAE(s). After discontinuation of PD-1 blockade, changes in HRQoL (FACT-M, EuroQol EQ-5D, CWS) will be measured at different time points and compared with patients without early discontinuation of PD-1 blockade. In addition, measurements will be performed to determine the impact of treatment discontinuation on healthcare resource use, productivity losses (RUQ-M by iMTA), and hours of informal care.

#### Control group

For statistical analyses, the results will be compared with a well-defined historical cohort of first-line treatment with pembrolizumab in advanced melanoma (KEYNOTE-006) [5]. In addition, data will be obtained from the DMTR, which prospectively registers clinical data and HRQoL of all patients with advanced and metastatic melanoma who are treated in the Netherlands [42].

### Sample size calculation

The sample size was estimated using the principles of an A-Hern design powered for 39% of ongoing response at 24 months. In order to be able to reject 29% under the alternative of 39% with a one-sided type I error of 5 and 90% power, 190 patients will be needed. Criterion for success according to the A’Hern design would be that at least 66 patients out of 190 patients continue to experience a response at 24 months after the first start of treatment. However, the primary test will be done using the lower boundary of the one-sided 95% CI of the Kaplan-Meier estimate of PFS from the start of treatment (which in this patient population is equivalent to an ongoing response) at 24 months to be able to account for possible drop-out or censoring before this time point. Taking a safety margin of 5% for lost to follow-up, the total sample size will be fixed at 200 patients.

### Risk analysis

Patients will be treated and evaluated according to the standard of care in the Netherlands. As PD-1 blockade will be discontinued earlier than two years, participation in this trial may affect treatment efficacy which will be evaluated as the primary objective of this study. As a result, participation in this trial may affect clinical outcome and even survival of these patients. Importantly, following the development of disease progression, re-treatment with monotherapy PD-1 blockade (preferred) or other treatment options can be initiated at the discretion of the treating medical oncologist. However, as an increasing number of physicians discontinue treatment on an individual basis at achieving CR or PR, the additional risk of participation in this trial is considered limited compared to daily clinical practice.

A Data Safety Monitoring Board (DSMB) will monitor quality and patient safety in this multicentre trial. As discontinuation of PD-1 blockade should neither lead to an increased number of fatal or life-threatening events, nor an increased incidence of new or symptomatic brain metastases, these are considered as SAEs which need to be reported expedited (i.e. within 24 h) throughout the course of the trial to the sponsor and will be made available to the DSMB. After the inclusion of 75 patients, the DSMB will review the data and advise on study continuation, based on the number and severity of SAEs. If the available data are not sufficiently mature after the inclusion of 75 patients (e.g. as a result of fast inclusion), 6-monthly DSMB evaluations will be added until the DSMB has confirmed that the provided data are mature, or until 200 patients are included. If the incidence of these particular SAEs is unexpectedly high, this could result in early termination of the study after consultation of the DSMB.

## Discussion

In this nationwide Safe Stop trial, the treatment regimen is optimised for individual patients with advanced melanoma by early discontinuation of first-line monotherapy with PD-1 blockers (i.e. nivolumab and pembrolizumab) upon achieving confirmed response (CR or PR) according to RECIST v1.1 [34]. All fourteen Dutch melanoma centres will participate to prospectively evaluate whether it is safe to stop first-line PD-1 treatment early. The primary endpoint is the rate of ongoing response, which will be assessed at 24 months after initial start of first-line monotherapy PD-1 blockade. To evaluate patients’ well-being, HRQoL will be collected periodically.

In this proof-of-concept study of early discontinuation of PD-1 blockade, maintaining patient safety is paramount and pursued by the study design in patients with a relatively favourable prognosis. The decision to discontinue treatment early is based on the treatment regimen and response-driven; only patients achieving CR or PR upon first-line monotherapy with PD-1 blockade are eligible. Patients with SD are not eligible, as patients who achieve SD as best overall response and complete two years of PD-1 blockade have a significantly worse PFS compared to patients with CR or PR (PFS of 40% vs. 82–85% at 24 months after completion of 2 year pembrolizumab treatment [5]). Currently, monotherapy with PD-1 blockade is the most frequently administered ICI for the treatment of advanced and metastatic melanoma in patients with a more favourable risk profile. In patients with rapidly progressive and/or (severe) symptomatic BRAF-mutant melanoma, BRAF-directed therapy is often prescribed as first-line treatment [8, 9]. In patients with rapidly progressive melanoma, cerebral metastases and/or elevated level of lactate dehydrogenase (LDH), combination therapy with nivolumab-ipilimumab is usually preferred, in particular in patients with BRAF-wild type melanoma. For the Safe Stop trial, patients treated with the combination nivolumab-ipilimumab or BRAF/MEK inhibitors will not be eligible, as inclusion of these patients would affect the homogenous population with regard to patient characteristics and administered therapy.

To ensure feasibility and nationwide implementation, the study was designed according to procedures of current clinical practice. First, patients treated with different PD-1 inhibitors (i.e. nivolumab or pembrolizumab) are eligible, as these drugs are considered interchangeable based on OS and AEs [5, 8, 22]. Second, as ^18^F-FDG-PET/CT is often performed for initial staging of melanoma, low-dose CT is allowed as baseline scan in strictly prespecified cases. Third, for response evaluation, RECIST instead of immune-based response criteria (iRECIST) is used, as iRECIST is not yet applied in daily clinical practice [34, 43]. Fourth, the study was not designed as randomised controlled trial to compare outcomes of patients with early discontinuation of PD-1 blockade with the outcomes of patients treated for two years. As patients usually have access to a treatment duration of two years, randomisation to early discontinuation of PD-1 blockade was considered not feasible in this setting. Furthermore, as an increasing number of physicians discontinue PD-1 blockade early on an individual basis [25], this trial design facilitates shared decision making and may prevent overtreatment, thereby potentially reducing AEs and the number of hospital visits. The decision to early discontinue PD-1 blockade in patients with tumour response is based on pre-specified criteria and patients’ wishes.

The Safe Stop trial also addresses the challenges physicians are facing during the current pandemic with coronavirus disease 2019 (COVID-19), including capacity issues in oncological care [44]. The design of the Safe Stop trial obviously contributes to a reduction in hospital visits and costs during this pandemic. Although it is yet not known whether patients treated with ICIs have a higher risk of (a severe course of) COVID-19, the Safe Stop trial may limit the risk of ICI associated AEs and thereby the use of immunosuppressive drugs. In contrast to other clinical trials, it has not yet been necessary to put the Safe Stop Trial on-hold during the COVID-19 pandemic, which is the result of its accessible trial design and additional value during this ongoing pandemic.

In the nationwide Safe Stop trial, we aim to investigate the proof of concept of early discontinuation of PD-1 blockade in melanoma patients treated with first-line monotherapy nivolumab or pembrolizumab upon achieving a tumour response. Potential advantages of treatment optimisation through early discontinuation include the prevention of overtreatment, thereby improving HRQoL, and reducing (S) AEs and healthcare costs.

## Data Availability

Data shall only be shared upon request with researchers who provide a methodologically sound research proposal, at the discretion of the principal investigator. Only de-identified participant data from the final research dataset used in the published manuscript can be shared. Results will be communicated via WIN-O, presentations at (inter)national conferences, and via publications in (peer-reviewed) journals.

## Abbreviations

AACR: American Association for Cancer Research
CI: Confidence interval
CR: Complete response
CT: Computed tomography
COVID-19: Coronavirus disease 2019
CWS: Cancer Worry Scale
DMTR: Dutch Melanoma Treatment Registry
DSMB: Data Safety Monitoring Board
ECCO: European Cancer Organisation
ESMO: European Society for Medical Oncology
EORTC: European Organisation for Research and Treatment of Cancer.
EQ-5D-5L: EuroQoL Health Utilities Index (EQ-5D, version 5L)
18F-FDG: Fluorine-18 fluorodeoxyglucose
FACT-M: Functional assessment of ancer therapy melanoma
ICI: Immune checkpoint inhibitor
iMTA: Institute for Medical Technology Assessment
iRECIST: Immunotherapy Response Evaluation Criteria In Solid Tumours
LDH: Lactate dehydrogenase
MRI: Magnetic resonance imaging
PD: Progressive disease
PD-1: Programmed cell death protein 1
PFS: Progression-free survival
PET: Positron emission tomography
PR: Partial response
(HR)QoL: (Health-related) Quality of life
RECIST v1.1: Response Evaluation Criteria In Solid Tumours version 1.1
(S)AE: (Serious) Adverse event
SD: Stable disease
Sponsor: The sponsor is the party that commissions the organisation or performance of the research (here: the academic hospital Erasmus Medical Centre Cancer Institute)
WIN-O: Werkgroep Immunotherapie Nederland voor Oncologie
